# Glucocorticoid induces human beta cell dysfunction by involving riborepressor GAS5 LincRNA

**DOI:** 10.1016/j.molmet.2019.12.012

**Published:** 2019-12-27

**Authors:** Jonathan L.S. Esguerra, Jones K. Ofori, Mototsugu Nagao, Yuki Shuto, Alexandros Karagiannopoulos, Joao Fadista, Hitoshi Sugihara, Leif Groop, Lena Eliasson

**Affiliations:** 1 Islet Cell Exocytosis, Department of Clinical Sciences-Malmö, Lund University, Malmö, Sweden; 2 Department of Endocrinology, Diabetes and Metabolism, Graduate School of Medicine, Nippon Medical School, Tokyo, Japan; 3 Department of Epidemiology Research, Statens Serum Institut, 2300, Copenhagen S, Denmark; 4 Diabetes and Endocrinology, Department of Clinical Sciences-Malmö, Lund University, Malmö, Sweden; 5 Lund University Diabetes Centre, Skåne University Hospital, Malmö, Sweden

**Keywords:** Glucocorticoid, Long intergenic non-coding RNA, Insulin secretion, Pancreatic islets, Beta cells, Type-2 diabetes mellitus

## Abstract

**Objective:**

A widely recognized metabolic side effect of glucocorticoid (GC) therapy is steroid-induced diabetes mellitus (DM). However, studies on the molecular basis of GC-induced pancreatic beta cell dysfunction in human beta cells are lacking. The significance of non-coding RNAs in various cellular processes is emerging. In this study, we aimed to show the direct negative impact of GC on beta cell function and elucidate the role of riborepressor GAS5 lincRNA in the GC signaling pathway in human pancreatic beta cells.

**Methods:**

Patients undergoing two weeks of high-dose prednisolone therapy were monitored for C-peptide levels. Human pancreatic islets and the human beta cell line EndoC-βH1 were incubated in pharmacological concentrations of dexamethasone. The GAS5 level was modulated using anti-sense LNA gapmeR or short oligonucleotides with GAS5 HREM (hormone response element motif). Immunoblotting and/or real-time PCR were used to assess changes in protein and RNA expression, respectively. Functional characterization included glucose-stimulated insulin secretion and apoptosis assays. Correlation analysis was performed on RNAseq data of human pancreatic islets.

**Results:**

We found reduced C-peptide levels in patients undergoing high-dose GC therapy. Human islets and the human beta cell line EndoC-βH1 exposed to GC exhibited reduced insulin secretion and increased apoptosis. Concomitantly, reduced expression of important beta cell transcription factors, PDX1 and NKX6-1, as well as exocytotic protein SYT13 were observed. The expression of the glucocorticoid receptor was decreased, while that of serum and glucocorticoid-regulated kinase 1 (SGK1) was elevated. The expression of these genes was found to significantly correlate with GAS5 in human islet transcriptomics data. Increasing GAS5 levels using GAS5 HREM alleviated the inhibitory effects of dexamethasone on insulin secretion.

**Conclusions:**

The direct adverse effect of glucocorticoid in human beta cell function is mediated via important beta cell proteins and components of the GC signaling pathway in an intricate interplay with GAS5 lincRNA, a potentially novel therapeutic target to counter GC-mediated beta cell dysfunction.

## Highlights

• Glucocorticoid (GC) causes direct human beta cell dysfunction.
• GC treatment and GAS5 reduction have negative effects on insulin secretion.
• GC treatment and GAS5 reduction result in perturbed expression of beta cell genes.
• The expression of GAS5 in human islets from diabetes donors is deregulated.
• Modulating GAS5 levels in beta cells can alleviate GC-mediated beta cell defect.

## 1 Introduction

Glucocorticoids (GCs) in various forms (hydrocortisone, dexamethasone, prednisolone, and prednisone) are highly potent steroid hormones in the frontline of various clinical therapy procedures. They are the most widely prescribed drugs used for treating allergic disorders, inflammatory and autoimmune diseases, some forms of malignancies, and suppressing immune response following organ transplantation [1,2]. Despite the efficacy of GC therapy in treating human diseases, metabolic side effects have been acknowledged, of which steroid-induced diabetes mellitus (DM) is the most recognized [3]. Indeed, rapid onset of hyperglycemia is observed in up to 80% of patients receiving high-dose GC treatment [4], and the incidence of new onset diabetes in these patients is estimated to be ≈ 50% [3,5,6].

The primary diabetogenic effect of GCs has been shown to be through impaired insulin signaling and deranged metabolic processes in liver, muscle, adipose, and bone tissues, collectively manifested as dyslipidemia, insulin resistance, and glucose intolerance [2,7]. The contribution of beta cell dysfunction to DM is well-established [8]. However, despite ample evidence of the direct effects of GCs in rodent beta cells [[9], [10], [11]], studies on the molecular basis of GC-induced pancreatic beta cell dysfunction in human beta cells are lacking.

An emerging complexity in gene regulation involves non-protein coding functional RNA molecules that can significantly influence diverse cellular processes. In pancreatic beta cells, the role of small RNAs such as microRNAs is now widely recognized [12], while the function of many long non-coding RNAs remains to be elucidated [13]. In glucocorticoid signaling, the non-coding RNA growth arrest-specific 5 (GAS5) acts as a GR riborepressor by directly interacting with the glucocorticoid receptor (GR) in a dexamethasone-dependent manner as demonstrated in HeLa cells [14]. However, the role of GAS5 in human pancreatic beta cell function has not been previously addressed.

In this study, we report on the deleterious effects of insulin secretion in at-risk patients undergoing chronic high-dose GC therapy, as opposed to augmented insulin secretion observed in GC-treated healthy individuals [15,16]. More importantly, we used human islets and the human beta cell line EndoC-βH1 to demonstrate the involvement of long intergenic non-coding RNA (lincRNA) GAS5 in GC-mediated beta cell dysfunction. Modulation of GAS5 in the human beta cell alleviated the GC-induced insulin secretion defect, demonstrating the potential of this non-coding RNA as a novel therapeutic target in countering GC-mediated beta cell dysfunction.

## 2 Materials and methods

### 2.1 Ethical statement

The patients in this study who underwent prednisolone therapy provided informed consent. This work was conducted in accordance with the Declaration of Helsinki for experiments involving humans. This study was approved by the ethics committee of Nippon Medical School Graduate School of Medicine, Tokyo, Japan. For experiments involving human and rodent pancreatic islets, all of the procedures complied with ethical permits issued by the Uppsala and Lund University Ethics committees and the Malmö/Lund Ethical Committee on Animal Research, respectively.

### 2.2 Prednisolone (PSL) treatment

On average, five patients received a total of 440 ± 208 mg of prednisolone within 11–17 days of treatment. On average, each patient received 33 mg of prednisolone per day. Their fasting serum C-peptide and fasting plasma glucose levels were monitored at three different time points: a day after starting PSL (on admission), a day after finishing PSL (on admission), and approximately 1 month after finishing PSL. The C-peptide index (CPI) was calculated as C-peptide (ng/mL) x 100/fasting plasma glucose (mg/dl). The patient characteristics, prednisolone doses, and length of therapy are provided in Supplementary Table 1.

### 2.3 Human pancreatic islets

Upon receipt of human pancreatic islets from Human Tissue Lab EXODIAB/LUDC via the Nordic Network for Islet Transplantation (http://www.nordicislets.org), they were handpicked under a stereomicroscope. The donors were grouped based on their glycated hemoglobin (% HbA1c) levels according to Fadista et al. [17]. We used pancreatic islets from normal glucose tolerant (NGT) donors (HbA1c < 6%; n = 10) and T2D donors (HbA1c ≥ 6.5%; n = 9) (Supplementary Table 2).

### 2.4 Rat pancreatic islets

Pancreatic islets were isolated from male Goto-Kakizaki and control Wistar rats as previously described by Esguerra et al. [18]. The animals were used at 8–15 weeks of age, at which point the non-fasting blood glucose levels of the GK rats (22.9 ± 1.6 mmol/L, N = 4) were significantly higher than those of the Wistar controls (6.3 ± 0.2 mmol/L, N = 4) (Student's t-test, two-sided, p < 0.001).

### 2.5 Cell culture

EndoC-βH1 cells (EndoCells, Paris, France) [19] were seeded in Matrigel fibronectin-coated (100 μg/mL and 2 μg/mL, respectively, Sigma–Aldrich, Steinheim, Germany) culture vessels in DMEM (Thermo Fisher Scientific, Waltham, MA, USA) containing 5.6 mM glucose, 2% BSA fraction V (Roche Diagnostics, Mannheim, Germany), 10 mM nicotinamide (Merck Millipore, Darmstadt, Germany), 50 μM 2-mercaptoethanol, 5.5 μg/mL transferrin, 6.7 ng/mL sodium selenite (Sigma–Aldrich), 100 U/mL penicillin, and 100 μg/mL streptomycin (PAA Laboratories, Pasching, Austria). The cells were incubated in a humidified atmosphere with 5% CO_2_ at 37 °C.

### 2.6 Silencing of lincRNA GAS5 by anti-sense LNA gapmeRs

EndoC-βH1 cells were seeded (180,000 cells per well) in a 48-well plate containing 150 μL medium without antibiotics (penicillin/streptomycin) a day before transfection. The following day, the cells were transfected with Exiqon's anti-sense LNA gapmeR targeting GAS5 (5′-3′ sequence: GAGTCCTTTGCTTCTT) or with the LNA gapmeR negative control A (5′-3′ sequence: AACACGTCTATACGC) using Lipofectamine RNAiMAX (Thermo Fisher). A final transfection volume of 200 μL per well contained 50 nM of the gapmeRs in Opti-MEM reduced serum media and 0.5 μL of Lipofectamine RNAiMAX (Life Technologies, San Francisco, CA, USA). The cells reached 90–100% confluence ≈72 h post-transfection and were assayed for insulin secretion and used for protein and RNA extraction as described to follow.

### 2.7 Transfection of GAS5 HREM RNA oligonucleotide

Using similar protocol as previously described to silence lincRNA GAS5, a control RNA oligonucleotide (RNA stem-loop) sequence that retains stem complementarity but lacks GAS5 HRE consensus (5′-CUGAUGGUCUUUGUAGACCAUCA-3′) and RNA GAS5 HREM (5′-CAGUGGUCUUUGUAGACUGCCUG-3′) was used (Integrated DNA Technologies, Coralville, IA, USA).

### 2.8 Dexamethasone treatment of human beta cells and islets

The cells and islets were treated with 2 μM dexamethasone (Dexa) with or without GR inhibitor RU486 and DMSO (vehicle, 1:1000) for 24 h for cells and 48 h for islets prior to GSIS. RU486 at 10 μM concentration was added at the same time as the dexamethasone.

### 2.9 Insulin secretion assay

Prior to insulin secretion, EndoC-βH1 cells were transferred to complete glucose starvation medium containing 2.8 mM for 18 h. Confluent plates were carefully washed twice with 1 mL pre-warmed secretion assay buffer (SAB), pH 7.2 (1.16 mM MgSO_4_, 4.7 mM KCl, 1.2 mM KH_2_PO_4_, 114 mM NaCl, 2.5 mM CaCl_2_, 25.5 mM NaHCO_3_, 20 mM HEPES, and 0.2% bovine serum albumin) containing 1 mM glucose. The cells were then pre-incubated in new 0.5 mL SAB with 1 mM glucose for 2 h. The cells were then stimulated for 1 h in 0.25 mL SAB with 1 mM glucose (basal secretion) or 20 mM glucose (GSIS) at 37 °C. Secreted insulin levels were measured using Mercodia Insulin ELISA #10-1113-01 (human) and the values were normalized to the total insulin content. Protein from each well was extracted using 100 μL RIPA buffer: 0.1% SDS, 150 nM NaCl, 1% Triton X-100, 50 mM Tris-Cl, pH 8, and EDTA-free protease inhibitor (Roche, Branchburg, NJ, USA). Protein content was analyzed using BCA assay (Pierce, Rockford, IL, USA) on Bio-Rad Model 6870 microplate reader.

For human islets, batches of five islets were pre-incubated for 30 min in 1 mM glucose and stimulated for 1 h with either 1 mM or 16.7 mM glucose in 0.5 mL of KREBS buffer (2.5 mM CaCl_2_, 4.7 mM KCl, 120 mM NaCl, 25 mM NaHCO_3_, 1.2 mM KH_2_PO_4_, 1.2 mM MgSO_4_, and 10 mM HEPES).

### 2.10 Total RNA extraction and quality control

Total RNA was extracted using Qiagen miRNeasy isolation kit according to the manufacturer's instructions (Qiagen, Hilden, Germany). The RNA concentration was measured using 1.5 μL on a NanoDrop (ND-1000, Thermo Fisher). The quality and integrity of the RNA were evaluated by both spectrophotometry and electropherogram profiles using NanoDrop (ND-1000) and Experion's automated electrophoresis system (Bio-Rad Laboratories, Hercules, CA, USA).

### 2.11 Quantification by real-time quantitative PCR (qPCR)

cDNA was generated from RNAs that passed the quality control test using a High Capacity cDNA Reverse Transcription kit according to the manufacturer's instructions (Applied Biosystems, Waltham, MA, USA). qPCR was performed in triplicate on a 384-well plate using an Applied Biosystems 7900HT standard RT-PCR system via default cycling parameters. Probe-based TaqMan Assays (Applied Biosystems) were used to measure the expression levels of human GAS5 (Hs03464472_m1), PDX1 (Hs00236830_m1), NKX6-1 (Hs00232355_m1), SYT13 (Hs00951871_m1), SGK1 (Hs00985033_g1), and GR (Hs00353740_m1). Hprt1 (4333768F) and PPIA (4333763F) were both used as endogenous controls. Relative quantification was conducted using the ΔΔCt method and the recalibrated values (2^−ΔΔCt^) were presented as the fold-change with respect to control or untreated conditions.

### 2.12 Western blotting analysis

Protein (15 μg homogenate) extracted after dexamethasone treatment or 72 h post-transfection was separated by 4–15% Mini-Protean TGX Precast gel from Bio-Rad Laboratories at 80 V. The protein was transferred to PVDF membranes, then blocked with 5% milk and 1% BSA in buffer consisting of 150 mM NaCl, 20 mM Tris-HCl, pH 7.5, and 0.1% (v/w) Tween for 1 h. The membranes were individually probed with antibodies against PDX1 (1:500, #2437, Cell Signaling Technology, Danvers, MA, USA), SYT13 (1:1000, # AP5482a, Abgent Inc., San Diego, CA, USA), NKX6-1 (1:200, ab129659, Abcam, Cambridge, UK), SGK1 (1:500, #12103, Cell Signaling Technology, Danvers, MA, USA), GR (1:500, #12041, Cell Signaling Technology, Danvers, MA, USA), cleaved caspase-3 (1:500, #9661, Cell Signaling Technology, Danvers, MA, USA), and Cyclophilin B (1:2000, ab16045, Abcam, UK) and incubated overnight at 4 °C. Horseradish peroxidase conjugated goat anti-rabbit IgG, HRP-linked antibody (1:10 000, #7074, Cell Signaling Technology, Danvers, MA, USA) was used to detect the primary antibodies. Clarity Western ECL Substrate and Bio-Rad ChemiDoc MP Imaging System (Bio-Rad Laboratories) was used to detect protein and protein quantification was done using Image Lab 5.2 software (Bio-Rad Laboratories).

### 2.13 Apoptosis assay

Apoptosis was assayed using a cell death detection kit according to the manufacturer's instructions (Roche) and validated by measuring the cleaved caspase-3.

### 2.14 Expression of GAS5 in pancreatic islets of type-2 diabetes donors and in Goto-Kakizaki rats

RNAseq data from 195 human islets. Partial data set available at Gene Expression Omnibus accession GSE50244 (n = 89) and GSE108072 (n = 88) were used for global co-expression analysis and correlation of GAS5 expression with donor phenotypes. A separate cohort of 19 human islet preparations from donors with T2D or controls were used for qPCR validation (Supplementary Table 3). Isolated islets from Goto-Kakizaki rats or Wistar controls were also used. The RNA or protein expression levels were determined by qPCR or Western blotting, respectively, as previously described.

### 2.15 Glucose treatment

The human beta cell line EndoC-βH1 cells were incubated in 5 mM glucose or 20 mM glucose for 1 h, 6 h, or 24 h. GAS5 expression at different time points was measured by qPCR.

### 2.16 Statistical analysis

All the data sets were tested for normality (Shapiro–Wilk's test) to determine the appropriate statistical tests as implemented in GraphPad Prism 7.04. Significant differences between control and GAS5 knockdown in insulin secretion, insulin content, and gene expression were tested using either Student's two-tailed or the non-parametric Mann–Whitney test. For multiple groups, significant differences were tested using one-way ANOVA (repeated measures) or the Kruskal–Wallis test followed by Dunnett's multiple comparison test (MCT) or Fisher's LSD. Two-way ANOVA was used to determine interactions between two factors. Data are presented as mean ± SEM.

## 3 Results and discussion

### 3.1 Glucocorticoid treatment reduces insulin secretion at high glucose concentrations and GAS5 expression in human islets and beta cells

**Figure 1:**
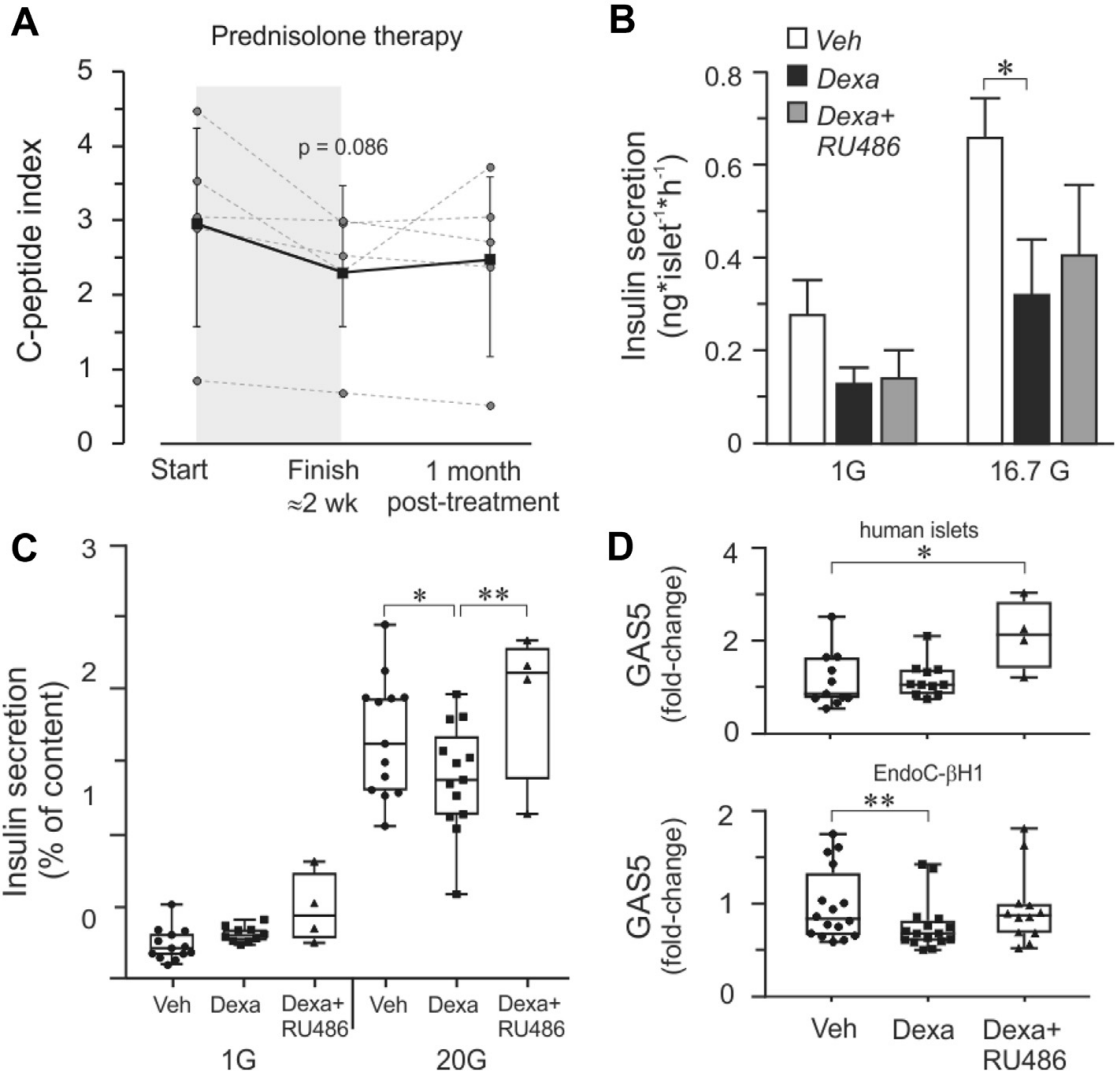
**Reduced insulin secretion and GAS5 expression upon glucocorticoid treatment. A.** C-peptide index (CPI) = C-peptide (ng/mL) x 100/fasting plasma glucose (mg/dL). Patients (n = 5) exhibit reduced trends in CPI on average (solid line) after prednisolone therapy. **B.** Dexamethasone-treated (Dexa) human islets display reduced insulin secretion at high glucose concentrations while the addition of GR inhibitor RU486 diminished the effect of dexamethasone. **C.** Insulin secretion measured in EndoC-βH1 beta cells. **D.** GAS5 expression in human islets and EndoC-βH1 cells. For all the experiments, the data are presented as an average of n = 4–16 biological replicates, mean ± SEM, *p < 0.05 and **p < 0.01. For insulin secretion, all of the comparisons between 1G and 20G were significantly different regardless of treatment but the symbols for significance were omitted for clarity.

The contribution of GC-mediated beta cell dysfunction to causing hyperglycemia was previously shown in vivo in which healthy individuals were subjected to acute or two-week prednisolone treatment resulting in reduced insulin secretion [15]. We found that the patients undergoing prednisolone therapy exhibited reduced fasting serum C-peptide levels by the end of an intensive high-dose two-week treatment period (Supplementary Fig. 1). However, while C-peptide per se denotes endogenous insulin levels, measuring it alone is insufficient to ascertain impaired beta cell secretion capacity. We therefore related the fasting serum C-peptide with the fasting serum glucose, which is the C-peptide index (CPI) shown to strongly predict future insulin use in T2D patients [20] and hence indicate impaired beta cell function [21]. The CPI showed reduced trends in all of the patients treated with prednisolone (Figure 1A).

Next, to determine whether glucocorticoid can directly influence human beta cell function, we incubated isolated human islets and the recently developed human beta cell line EndoC-βH1 [19] in pharmacological concentrations of dexamethasone for 24 or 48 h and found significant reductions in insulin secretion (GSIS) at high glucose concentrations (Figure 1B,C). The total insulin content was assessed in all the insulin secretion experiments involving the cell line and no significant differences were observed regardless of treatment (Supplementary Fig. 2).

The mode of action of GCs has been extensively investigated in the context of inflammatory and immunomodulatory response. GCs act as ligands that bind the cytosolic glucocorticoid receptor (GR), which is then translocated into the nucleus and interacts within DNA promoter regions, specifically binding into a sequence motif called “glucocorticoid response element” (GRE) or negative GRE sites (nGRE), leading to either activation or inactivation of genes [[22], [23], [24]]. A widely used GR antagonist is compound RU486. In its presence, we observed partially diminished effects of dexamethasone on GSIS in both the human islets and EndoC-βH1 cells, although complete recovery was more apparent in the beta cell line (Figure 1B,C).

Another regulator of glucocorticoid signaling is non-coding RNA growth arrest-specific 5 (GAS5), which directly interacts with GR at its DNA-binding domain in a dexamethasone-dependent manner, hence acting as a GR riborepressor [14]. Remarkably, human GAS5 gene sequence is not fully conserved compared to mouse Gas5 due to an AluJo insertion, and the Gas5 steroid receptor recognition sequence is conserved only among primate-clade haplorhines [25]. GAS5 lincRNA as a tumor suppressor has mostly been studied in the cancer field [26]. Interestingly, among the 493 lincRNAs we compiled from RNAseq data of 89 human pancreatic islet preparations [17], we found GAS5 to be most highly expressed (Supplementary Table 3). GAS5 expression itself appears to be regulated within the GC-GR pathway since exposure of EndoC-βH1 human beta cells to dexamethasone resulted in reduced GAS5 levels, which recovered in the presence of GR inhibitor RU486 (Figure 1D). However, in human islets, we did not observe a significant reduction in GAS5 upon dexamethasone treatment possibly due to the contribution of other endocrine islet cell types in GAS5 expression. Indeed, mining the single-cell RNAseq data of the pancreas reveals almost equal levels of GAS5 in alpha and beta cells, which is also present in other endocrine and even non-endocrine cell types (http://sandberg.cmb.ki.se/pancreas/) [27].

### 3.2 Reduced GAS5 expression in beta cells leads to impaired insulin secretion and increased apoptosis

**Figure 2:**
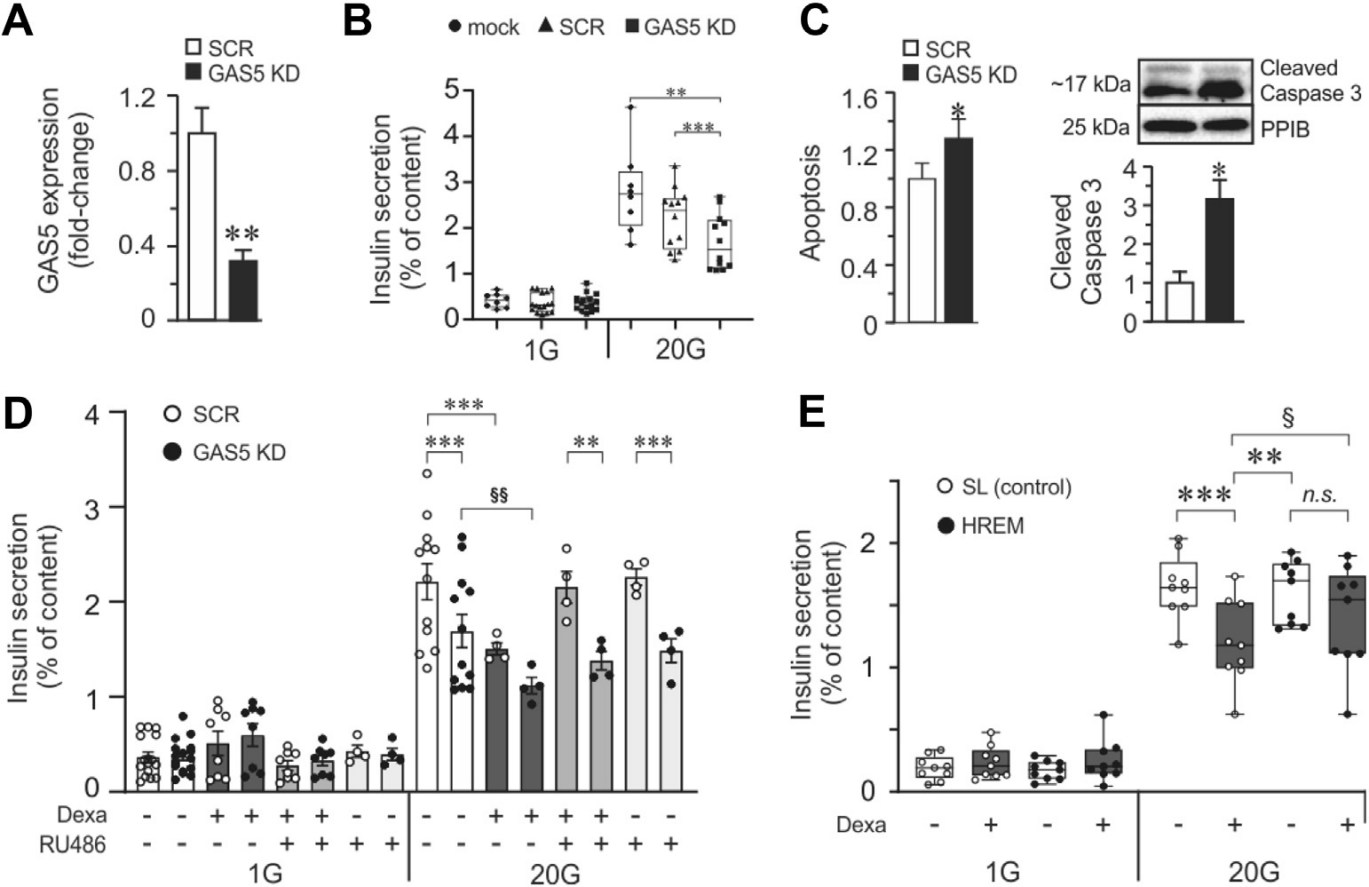
**Effect of GAS5 knockdown in beta cells and rescue by GAS5 HREM. A.** GAS5 expression is reduced to ≈40% after transfecting cells with LNA gapmeR against GAS5. **B.** GSIS assay upon GAS5 knockdown in EndoC-βH1 cells. **C.** Measurement of apoptosis upon GAS5 knockdown. **D.** GSIS measurement upon GAS5 KD in combination with dexamethasone treatment in EndoC-βH1 with or without RU486. **E.** Rescue of GSIS by GAS5 hormone response element mimic (HREM) in GC-treated cells. The stem-loop (SL) sequence from GAS5 is non-functional and is used as a negative control. For all the experiments, the data are presented as an average of n = 4–16 biological replicates, mean ± SEM, §, *p < 0.05 and §§, **p < 0.01.

To investigate whether reduced GAS5 during dexamethasone treatment contributes to impaired GSIS, we knocked down GAS5 using LNA gapmeRs against GAS5. We attained >60% reduction in GAS5 in the EndoC-βH1 cells (GAS5 KD cells) (Figure 2A), which resulted in both impaired insulin secretion at a high glucose concentration (20 mM) (Figure 2B) and increased apoptosis (Figure 2C). GAS5 knockdown or dexamethasone treatment resulted in similar insulin secretion defects at high glucose stimulation, implying convergent roles in the stimulated secretion coupling in the beta cells (Figure 2D). However, RU486 could rescue only the cells treated with dexamethasone alone and not the GAS5 KD cells. Moreover, GAS5 KD was more potent in eliciting negative effects on GSIS than just by dexamethasone treatment or in combination. This can be explained by the GAS5 levels being more reduced in the LNA gapmeR transfected cells than that when the cells were treated only with dexamethasone.

### 3.3 Combined negative effects of reduced GAS5 and glucocorticoid on insulin secretion are alleviated by GAS5 HREM treatment

We observed the combined negative effects of downregulated GAS5 and dexamethasone on insulin secretion in cells stimulated with 20 mM glucose, in which GAS5 KD cells treated with dexamethasone displayed significantly lower secretion than GAS5 KD cells (Figure 2D, comparison denoted by the symbol for significance, §).

When we introduced the active segment of GAS5 called the hormone response element mimic (HREM) [28] into the beta cells, we observed recovery of insulin secretion in dexamethasone-treated cells compared to stem-loop (SL) control cells also incubated in dexamethasone (Figure 2E), confirming the direct role of GAS5 in mediating the negative effect of GCs in beta cell GSIS.

### 3.4 Glucocorticoid treatment or GAS5 downregulation impacts the expression of key proteins in GC signaling and beta cell function

**Figure 3:**
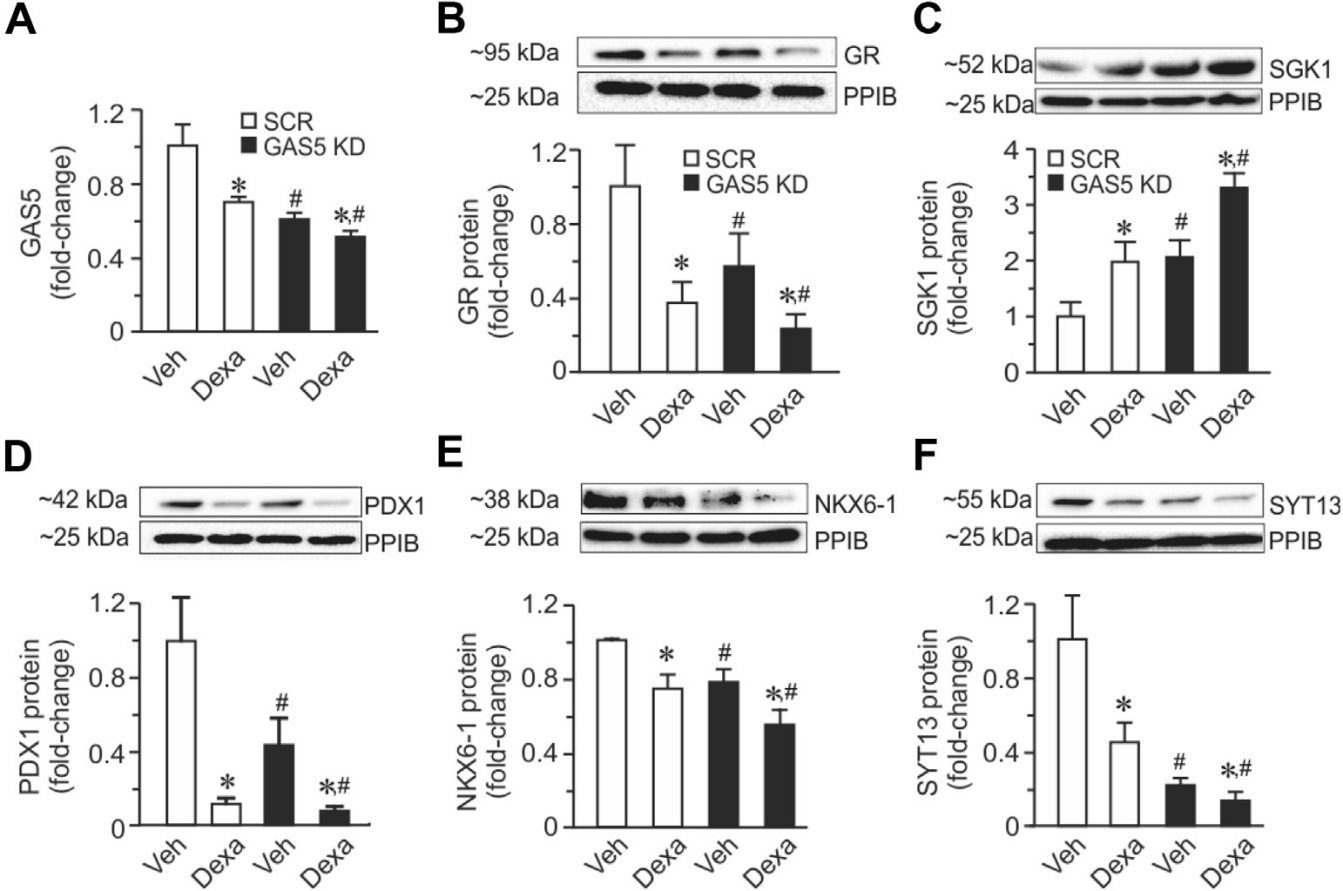
**GAS5 KD with or without GC treatment affects the expression of key proteins in glucocorticoid signaling (GR and SGK1) and beta cell function (PDX1, NKX6-1, and SYT13). A.** Expression of GAS5. **B.** Protein expression of GR. **C.** Protein expression of SGK1. **D.** Expression of PDX1. **E.** Protein expression of NKX6-1. **F.** Protein expression of SYT13. The data are presented as mean ± SEM. n = 4, *p < 0.05 vehicle vs Dexa; ^#^p < 0.05 scramble vs GAS5 KD.

To elucidate the potential co-regulation of GAS5 non-coding RNA with other protein-coding genes in the human islets, we performed co-expression analyses using RNAseq data. We observed significant negative correlations of GAS5 RNA with the mRNA levels of transcription factors PDX1 and NKX6-1, the exocytotic gene synaptotagmin 13 (SYT13), GR, and positive correlation with SGK1 (Supplementary Fig. 3). PDX1 is a transcription factor essential for both beta cell differentiation and insulin secretion, while NKX6-1 is essential for insulin biosynthesis, secretion, and beta cell proliferation [29]. SYT13 is a member of the synaptotagmin protein family that is possibly involved in the transport of insulin vesicles docking to the plasma membrane. We showed in human islets that SYT13 expression correlated negatively with donor HbA1c measurements while correlating positively with GSIS, results that were further corroborated with reduced GSIS in INS-1 832/13 cells upon SYT13 knockdown [30]. In beta cells, GC has been reported to cause beta cell dysfunction via GR, leading to impaired insulin secretion [2,[31], [32], [33]]. It has also been demonstrated that GCs directly inhibit GSIS in vivo, making the beta cells among the important targets for diabetogenic action of GCs [34]. Abnormal increased expression of SGK1 in pancreatic beta cells leads to upregulation of voltage-sensitive Kv1.5 channels, hyperpolarization, and reduced Ca^2+^ entry via voltage-gated Ca^2+^ channels, contributing to impaired insulin secretion observed upon GAS5 knockdown [11,[35], [36], [37]]. In this study, we showed that both GAS5 KD or dexamethasone treatment resulted in reduced GAS5 levels (Figure 3A), leading to concomitant reductions in GR, PDX1, NKX6-1, and SYT13 proteins (Figure 3B,D, E, and F) and increases in SGK1 protein (Figure 3C). The effect of dexamethasone treatment on the expression of GR, SGK1, PDX1, NKX6-1, and SYT13 in both human islets and EndoC-βH1 cells were reversed in the presence of the GR inhibitor RU486 (Supplementary Fig. 4), confirming the regulation of these genes via GC-GR signaling. Taken together, we conclude that GC-mediated downregulation of GAS5 in beta cells may lead to downregulation of proteins important for beta cell function and hence negative effects on GSIS.

### 3.5 Dysregulated expression of GAS5 and correlated proteins in islets from T2D donors and GK rats under glucotoxic conditions

**Figure 4:**
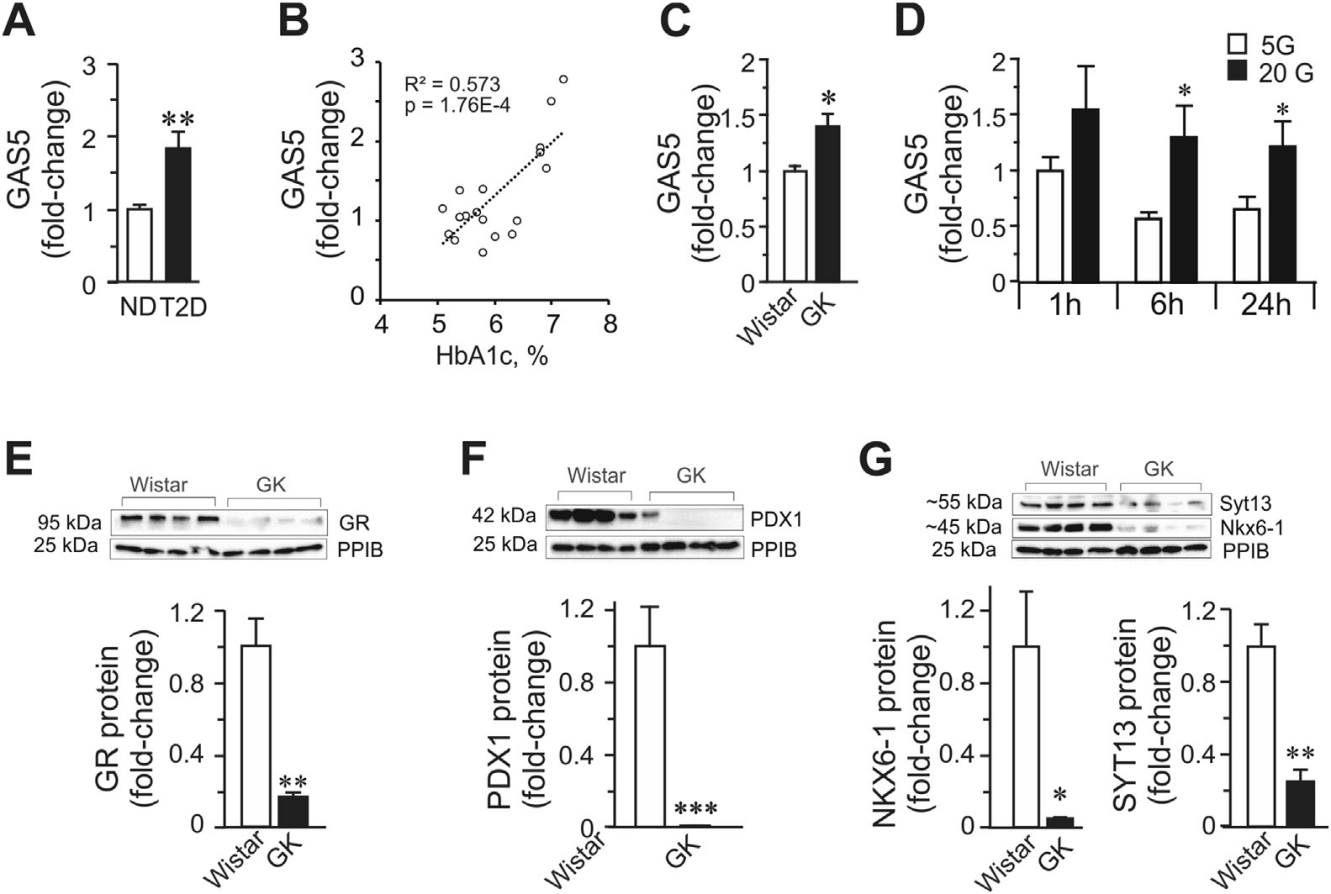
**GAS5 and gene expression changes in islets from diabetic donors, GK islets, and beta cells under glucotoxic conditions. A.** Expression of GAS5 in islets from control (ND) (n = 10) and T2D donors (n = 9) as measured by qPCR assay. **B.** GAS5 expression in islets vs HbA1c levels in all of the donors (n = 19). **C.** GAS5 expression in the islets of T2D model GK rats. **D.** Glucose regulation of GAS5 in EndoC-βH1 cells (n = 4) exposed to glucotoxic conditions. **E.** GR protein expression. **F.** Protein expression of PDX1. **G.** Protein expression of NKX6-1 and SYT13. The data are presented as mean ± SEM; *p < 0.05, **p < 0.01, ***p < 0.001.

The importance of functional non-coding RNA in various cellular processes is now widely recognized. In the field of cancer, many lncRNAs have been shown to be dysregulated and some, such as GAS5 lincRNA, have been ascribed functions relevant to the development of disease. For instance, as a tumor suppressor, GAS5 was expectedly found to be markedly decreased in multiple human cancer types [26]. To examine how GAS5 is expressed under glucotoxic conditions, we compared GAS5 expression under the following conditions: i) human islets from controls vs T2D donors (Figure 4A,B), ii) islets from the type-2 diabetes (T2D) animal model Goto-Kakizaki (GK) rats vs control Wistar rats (Figure 4C), and iii) EndoC-βH1 beta cells incubated in 5 mM glucose or 20 mM glucose at three different time points (Figure 4D). We found that in all these instances, the GAS5 expression level was elevated in hyperglycemic environments. Notably, a positive correlation between the long-term glycemia in the donors and GAS5 islet expression was observed (Figure 4B), indicating the continuous upregulation of GAS5 in response to increasing glucose levels in the blood. The observed upregulation of GAS5 in pancreatic islets and human EndoC-βH1 beta cells under a hyperglycemic environment was surprising given the results of our GAS5 KD experiments in which we observed impaired GSIS due to GAS5 downregulation. We therefore hypothesize that the upregulation of GAS5 is a component of beta cell adaptation response to hyperglycemia, presumably a compensatory mechanism with the aim of influencing the GC-GR pathway to prevent beta cell failure. Nonetheless, the upregulation of GAS5 is not sufficient to prevent the demise of important beta cell factors (Figure 4E,F, and G) that are susceptible to glucotoxic environments ultimately leading to beta cell dysfunction.

## 4 Conclusions

In conclusion, we showed that glucocorticoid-induced dysfunction in human EndoC-βH1 beta cells may act partially through GAS5-mediated regulation of the GC-GR pathway, in which GR and SGK1 play major roles in regulating downstream target genes, most likely through the beta cell transcription factors PDX1 and NKX6-1. We demonstrated that dexamethasone treatment and downregulated GAS5 levels can separately affect the expression of proteins involved in the GC-GR pathway, ultimately converging to impaired insulin secretion in beta cells exposed to stimulating glucose concentration. We observed a reduction in the GAS5 levels upon dexamethasone treatment and a combined negative effect (but not synergistic) of both glucocorticoid and knockdown of GAS5 on insulin secretion. This suggests partial overlap of pathways in which dexamethasone treatment and reduced GAS5 levels can separately mediate impaired insulin secretion, implying that these two processes may not simply be linearly connected. We cannot, however, at this time conclusively extend our results to primary human beta cells due to difficulty in acquiring human pancreatic islets and the technical challenge of sorting beta cells for subsequent functional studies.

Another limitation of the current study is the lack of direct investigation of how glucocorticoid and GAS5 mediates beta cell dysfunction in pancreatic islets from donors with steroid-induced diabetes mellitus. Procurement of human islet material poses a considerable challenge, while rodent models have inherent limitations when it comes to investigating non-fully conserved genetic elements such as non-coding RNA genes.

Nonetheless, we demonstrated the therapeutic potential of manipulating GAS5 activity in beta cells using functional GAS5 HREM to rescue GC-induced impairment of GSIS. Considering the emerging field of RNA-based therapeutics, our identification of the involvement of GAS5 lincRNA in GC-induced pancreatic beta cell dysfunction may be a novel therapeutic target in patients undergoing glucocorticoid therapy to improve beta cell function.

## Author contributions

J.L.S.E., J.O., M.N., and L.E. designed this study. J.L.S.E, J.O., M.N., S.Y., A.K., and J.F. conducted the research. J.L.S.E, J.O., M.N., S.Y., A.K., J.F., H.S., L.G., and L.E. analyzed the data. J.L.S.E., J.O., M.N., and L.E. wrote the paper. All of the authors reviewed the manuscript.
