# Supplementary material for: Glucocorticoid induces human beta cell dysfunction by involving riborepressor GAS5 LincRNA
Source: Mol Metab. 2019 Dec 27;32:160–7. doi: 10.1016/j.molmet.2019.12.012 (PMC6976904; doi:10.1016/j.molmet.2019.12.012)
Supplement: Multimedia component 1 [file mmc1.docx]

**Appendix A. Supplementary data**

**Supplementary figures**


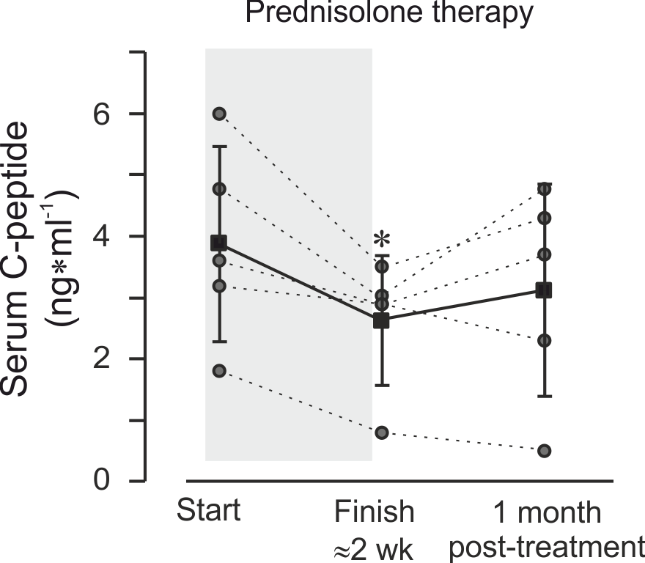


**Supplementary Figure 1**

Patients (n = 5) exhibited reduced fasting serum C-peptide on average (solid line) after prednisolone therapy. Student’s t-test (paired, two-tailed) *p < 0.05.


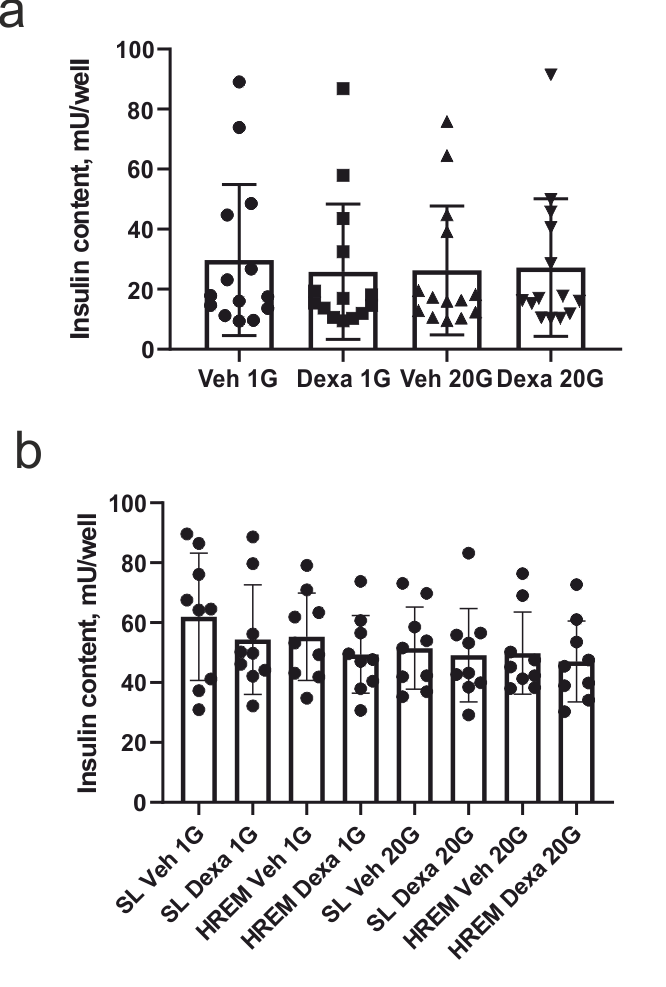


**Supplementary Figure 2. Example of insulin content measurements used to normalize insulin secretion during glucose-stimulated insulin secretion assays**

No significant differences in insulin content were observed when EndoC-BH1 cells were treated with **a.** dexamethasone at low or high glucose (corresponding to GSIS in Fig. 1c) or **b.** transfected with GAS5 stem-loop (SL) control or GAS5 hormone-responsive element motif (HREM) oligonucleotide under different conditions (corresponding to GSIS in Fig. 2e). (Veh, vehicle; Dexa, dexamethasone; 1G, 1 mM glucose; 20G, 20 mM glucose).

**
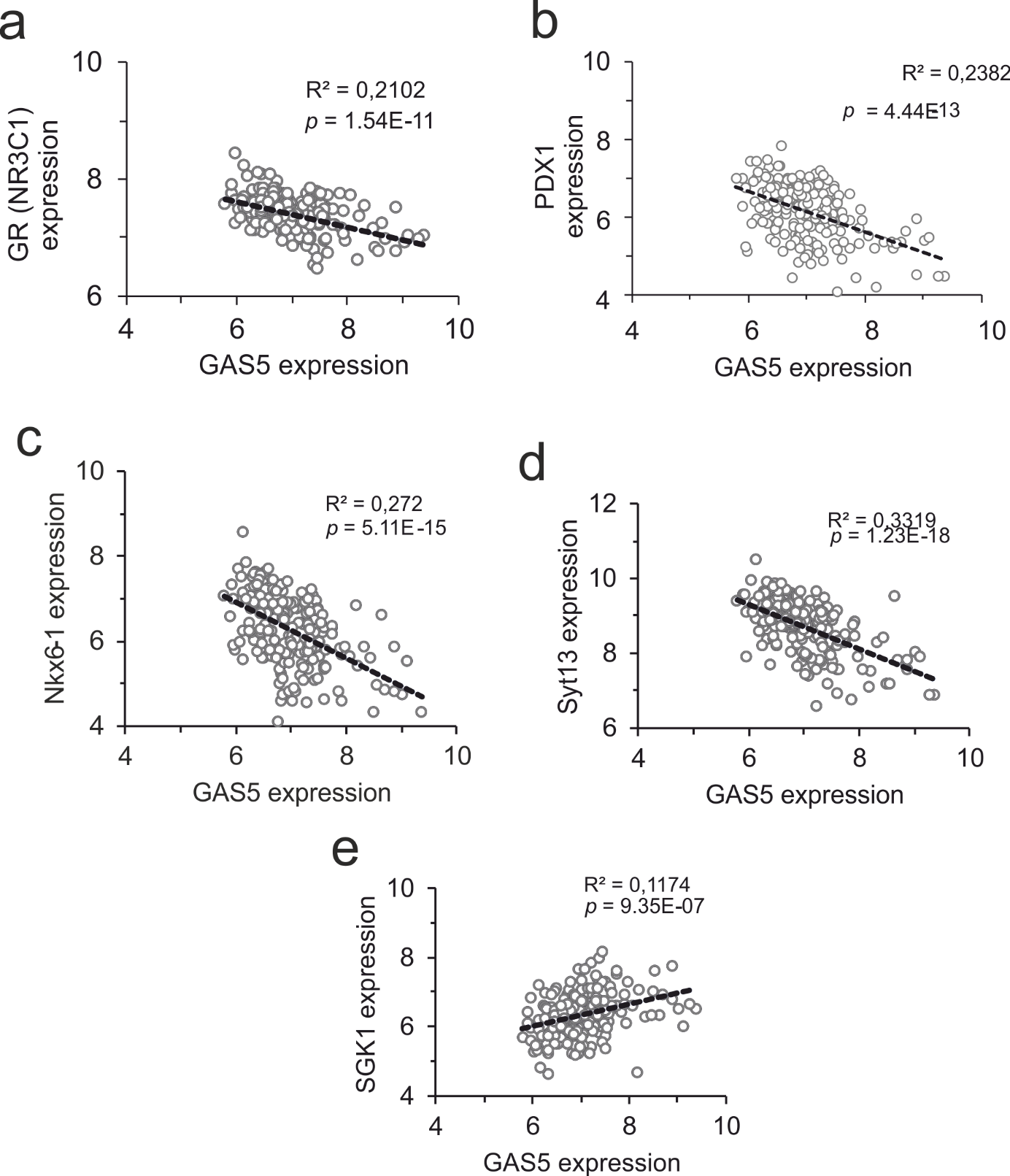
**

**Supplementary Figure 3. Co-expression analyses of GAS5 in RNAseq data from human pancreatic islet preparations (N = 195 donors)**

GAS5 mRNA levels negatively correlated with those of **a.** GR (NR3C1), **b.** PDX1, **c.** NKX6-1, and **d.** SYT13 and positively correlated with the expression of **e.** SGK1*.*

**
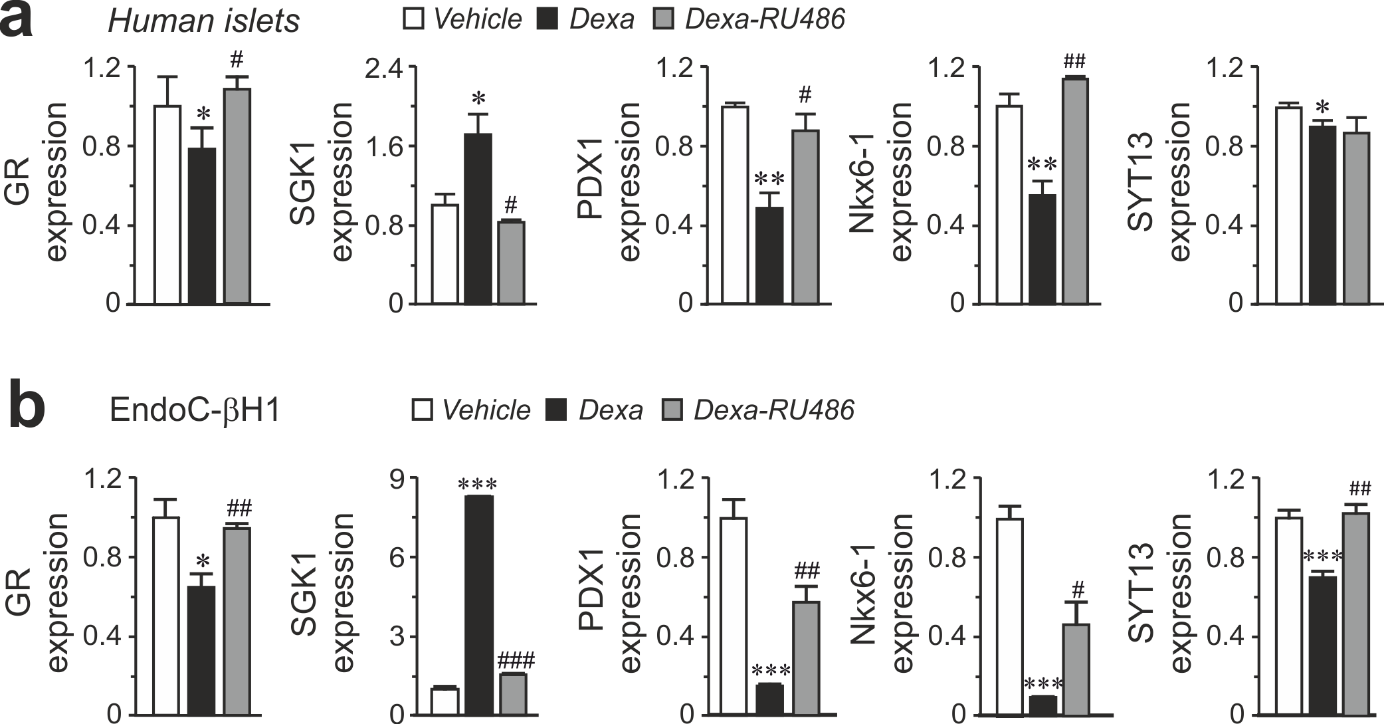
**

**Supplementary Figure 4. mRNA expression of GAS5-correlated genes upon glucocorticoid treatment in human islets and EndoC-βH1 cells**

**a.** Expression of GR, SGK1, PDX1, NKX6-1, and SYT13 in human islets **b.** Expression of GR, SGK1, PDX1, NKX6-1, and SYT13 in EndoC-βH1 cells. The data are presented as an average of n = 4-5 biological replicates, mean ± SEM, *p < 0.05 and **p < 0.01, ***p < 0.001 vs vehicle; ^#^p < 0.05; ^#,#^p < 0.01 and ^#,#,#^p < 0.001 vs Dexa.

**Supplementary tables**

| **Supplementary Table 1. Characteristics of the patients who underwent prednisolone therapy** | | | | | | | |  |  |
| --- | --- | --- | --- | --- | --- | --- | --- | --- | --- |
|  |  |  |  |  |  |  |  |  |  |
| **Patient** | **Age** | **Sex** | **Disease** | **Duration of diabetes** | **Total PSL amount (mg)** | **Duration (days)** | **Start (1 day after starting PSL)** | **Finish (1 day after finishing PSL)** | **After 1 month** |
| 1 | 52 | F | Facial palsy | None | 330 | 12 | None | Insulin | Insulin |
| 2 | 45 | M | Facial palsy | 5 years | 800 | 17 | Diet | Insulin, saxagliptin | Saxagliptin |
| 3 | 63 | F | Sudden deafness | 5 years | 270 | 11 | Diet | Insulin, metformin | Metformin |
| 4 | 55 | F | Sudden deafness | None | 405 | 15 | None | Insulin | Insulin |
| 5 | 77 | M | Sudden deafness | None | 415 | 11 | None | Insulin | Diet |

| **Supplementary Table 2. Characteristics of the human islet donors** | | | | | |
| --- | --- | --- | --- | --- | --- |
|  |  |  |  |  |  |
| **Islet ID** | **BMI** | **Gender** | **Age** | **HbA1c** |  |
| CTRL 1 | 26.2 | Male | 73 | 5.4 |  |
| CTRL 2 | 28.4 | Male | 54 | 5.8 |  |
| CTRL 3 | 21.5 | Female | 68 | 5.2 |  |
| CTRL 4 | 25.7 | Female | 53 | 5.5 |  |
| CTRL 5 | 23.7 | Male | 57 | 5.3 |  |
| CTRL 6 | 25.7 | Female | 58 | 5.1 |  |
| CTRL 7 | 33.4 | Female | 49 | 5.8 |  |
| CTRL 8 | 24.9 | Male | 51 | 5.4 |  |
| CTRL 9 | 27.8 | Female | 49 | 5.7 |  |
| CTRL 10 | 29.9 | Male | 57 | 5.4 |  |
| T2D 1 | 29.4 | Female | 44 | 6.9 |  |
| T2D 2 | 22.5 | Male | 41 | 6.8 |  |
| T2D 3 | 24.5 | Male | 58 | 5.8 |  |
| T2D 4 | 34.6 | Female | 57 | 6.8 |  |
| T2D 5 | 28.7 | Female | 43 | 7 |  |
| T2D 6 | 34.9 | Male | 62 | 6.4 |  |
| T2D 7 | 28.5 | Female | 56 | 6 |  |
| T2D 8 | 27.8 | Male | 65 | 7.2 |  |
| T2D 10 | 22.9 | Female | 67 | 6.3 |  |

| **Supplementary Table 3. Top highly expressed lincRNAs in human islets (N = 89) compiled by Fadista et al., PNAS 2014** | |
| --- | --- |
|  |  |
| **Gene symbol** | **Normalized mean expression (arbitrary unit)** |
| GAS5 | 22.279 |
| SNHG5 | 9.042 |
| NCRNA00188 + SNORD49B | 7.042 |
| NME1 + NME2 + NME1-NME2 | 4.051 |
| NCRNA00275 | 2.205 |
| SNHG4 + MATR3 | 2.109 |
| C19orf30 | 1.963 |
| NEAT1 | 1.946 |
| SNHG8 | 1.821 |
| SNHG6 | 1.631 |
| GLG1 | 1.519 |
| MALAT1 | 1.178 |
| KIAA0114 | 1.008 |
| NCRNA00263 | 0.966 |
| SNHG1 | 0.740 |
| MEG3 | 0.739 |
